# Gluten and its relationship with inflammation and Parkinson's Disease: A literature review

**DOI:** 10.3934/Neuroscience.2025004

**Published:** 2025-03-31

**Authors:** Zohaer Muttalib, Dana Aboukhalil, Chisom Nwosu, Dave D. Manguerra, Jimmy Wen, Ubaid Ansari, Meraj Alam, Ihab Abed, Ethan Tabaie, Ahmed Salem, Forshing Lui

**Affiliations:** Department of Neurology, California Northstate University College of Medicine, 9700 W Taron Drive, Elk Grove, CA 95757, USA

**Keywords:** Parkinson's Disease, gluten, inflammation, dystonia, dyskinesia, Substantia Nigra

## Abstract

Parkinson's Disease is a neurodegenerative central nervous system (CNS) disease that primarily affects the dopaminergic cells of the Substantia Nigra in the midbrain and causes a diverse array of symptoms, including dystonia, a loss of balance, difficulty initiating movements, akinesia, muscle spasms, and tremors. It has long been known that environmental and commercial compounds are linked to an increased risk of Parkinson's Disease. Of importance, gluten, a complex polysaccharide, has been hypothesized to cause some of the symptoms related to Parkinson's Disease. It is hypothesized that gluten causes a chronic inflammatory state which may lead to plaque formation and neuronal cell death in the substantia nigra, alongside the symptoms of Parkinson's Disease. This literature review hopes to explore the relationship gluten has as an inflammatory molecule and its role in the production and prolongation of the disease processes in Parkinson's Disease.

## 1. Introduction

Parkinson's Disease is a neurodegenerative disease of the central nervous system (CNS) that causes both motor and non-motor symptoms. The symptoms are mainly tied to the damage of the Substantia Nigra of the midbrain and the development of neuronal alpha-synuclein deposition in this region. The Substantia Nigra supplies dopamine to the basal ganglia, which allows for voluntary muscle control and the smoothness of movements. Nerve cell death of the Substantia Nigra leads to the characteristic motor features of Parkinson's Disease, which include muscle rigidity, postural instability, tremors, and bradykinesia. Additionally, Parkinson's Disease has several non-motor symptoms, such as anosmia, constipation, sleep disorders, and the following neuropsychiatric disorders: depression, anxiety, and impulse control disorders. Parkinson's Disease has been known to occur due to several reasons, including genetic, environmental toxin exposures, and even Traumatic Brain Injuries (TBIs).

The environmental causes of Parkinson's Disease have been documented for many decades. Some of the best-studied causes are pesticides, which include but are not limited to Paraquat, Rotenone, Benomyl, and Mancozeb, which have been reported to cause 1 in 5 cases of Parkinson's Disease [1]. Other causes, such as the organic drug 1-methyl-4-phenyl-l,2,3,6-tetrahydropyridine (MPTP), were found to significantly increase the risk, as per the 1982 experiment, where 7 volunteers voluntarily injected themselves with MPTP and developed severe, irreversible end-stage Parkinson's Disease [2]. Trichloroethylene (C2HCl3), a metal degreasing solvent, was also linked to an increased incidence of Parkinson's Disease [3]. Heavy metals, such as iron (Fe), mercury (Hg), manganese (Mn), copper (Cu), and lead (Pb), have also been linked to Parkinson's Disease, though through an indirect pathway. Exposure to these metals leads to the chronic activation of inflammatory cytokines, which are hypothesized to lead to the deposition of alpha-synuclein and the development of Parkinson's Disease [4].

Gluten is a structural protein found in wheat composed primarily of gliadin and glutenin. It is a known inflammatory molecule that can trigger adverse immunological and autoimmune reactions in people with Celiac Disease (CD) (1%–2% of the general population) and non-celiac gluten sensitivity (1%–13% of the general population) [5]. Gluten has also been implicated in Gluten Ataxia, which is a rare neurological disease associated with gluten consumption that leads to an autoimmune response that targets the cerebellum and leads to gait and balance issues [6]. Neurological issues such as cerebellar ataxia, peripheral neuropathy, dementia, and neuropsychiatric disorders have been linked to CD, thus suggesting that gluten-induced inflammation may contribute to the disruption of the blood-brain barrier (BBB) integrity [7]–[9]. Additionally, increased BBB permeability, often caused by inflammation or bacterial activity, has been associated with various neurological conditions, including autism spectrum disorder (ASD), dementia, Alzheimer's disease, schizophrenia, and Parkinson's Disease [10],[11].

Although gluten intolerance and sensitivity are significant concerns, it is essential to remember that these diseases make up a small portion of the total patient population in the United States. Therefore, patients with predispositions to these gastrointestinal conditions may more specifically be affected by gluten and inflammation, which may lead to the development of neuroinflammation and neurological diseases. It may be more difficult to correlate inflammation to patients that do not have these reactions to gluten and may differentially affect these patients. This paper will explore the connection between gluten as an inflammatory molecule in at-risk patients and its potential connection with the development of Parkinson's Disease.

## 2. Parkinson's Disease

Parkinson's Disease was first described by English physician Dr. James Parkinson in 1817 and was characterized as a “shaking palsy” that initially began on a single extremity and spread to the rest of the extremities and the head. Although Dr. Parkinson's assessment and physical exam findings were correct, further research over the past two centuries has helped us to better understand the symptoms, pathophysiology, and neuroanatomy of Parkinson's Disease. Parkinson's Disease is the second most common neurodegenerative disease after Alzheimer's Disease and has a prevalence of 0.5%–1% among the 65–69-year-old population and 1%–3% in the 80 years and older population. An incidence rate of 90,000 new patients every year was reported in 2022. Although Parkinson's normally affects older patients, there is also a subset of patients who are diagnosed earlier than the usual age, and these patients are usually termed Early Onset Parkinson's Patients. These patients are hypothesized to have a genetic cause which leads to the formation of Parkinson's Disease earlier than the typical age range. Moreover, these patients are reported to have a faster disease onset and progression, the prevalence of certain symptoms, and the absence of others.

Parkinson's Disease classically has a slow onset of disease but is progressive and leads to irreversible damage of the substantia nigra, which is the main dopaminergic region of the brain. The substantia nigra is a small, darkly pigmented area of the midbrain that produces essential dopamine for several neurological functions. The substantia nigra is comprise of the Pars Compacta, which contains dopamine-producing cells, and the Pars Externa, which helps coordinate movements. Parkinson's Disease is characterized by the loss of the nigrostriatal dopaminergic pathway, which connects the substantia nigra to the striatum. The loss of this pathway causes difficulty initiating movements and performing smooth muscle control. Recent studies have shown that the loss of the dopamine-producing cells of the Pars Compacta is preceded by a loss of the axons connecting the substantia nigra to the striatum [12]. The gross pathology of neurons from the Substantia Nigra region shows intracellular alpha-synuclein plaque lesions, which are termed Lewy Bodies. It is thought that the deposition of these Lewy Bodies possibly due to inflammation can lead to the death of the Substanitia Nigra neurons and Parkinson's Disease presentation. Mitochondrial dysfunction is also considered a key element of both idiopathic and familial Parkinson's Disease. Postmortem studies of the brains of Parkinson's Disease patients have shown that some patients lacked mitochondrial complex I, which is essential for the electron transport chain (ETC).

Parkinson's Disease is characterized by prodromal or pre-motor symptoms, which usually occur 12–14 years before disease presentation. One possible hypothesis of the beginning of the disease is the accumulation of misfolded proteins in the autonomic nervous system and the olfactory bulb, moving upwards via the vagus nerve to the CNS [13]–[15]. This may explain some of the early signs of Parkinson's Disease, which include constipation, hyposomia, and rapid eye movements (REM) before the development of motor symptoms. One study showed that patients with tremors, balance problems, depression, constipation, fatigue, and urinary dysfunction 5 years before diagnosis were more likely to develop PD than those without these symptoms [16]. The motor symptoms of Parkinson's Disease include the presence of bradykinesia, rigidity, tremor, difficulty walking, or difficulty of making facial expressions. Early symptoms generally present asymmetrically, with the absence of atypical symptoms (cerebellar signs, early severe autonomic dysfunction, vertical supranuclear palsies, or cortical sensory loss), which would be indicative of an alternative diagnosis [17]. Some of the non-motor symptoms of Parkinson's Disease include depression, anxiety, and mood disorders. It is thought that these psychiatric issues occur due to changes in dopamine and other neurotransmitters, which lead to these symptoms.

Parkinson's Disease is treated with several dopamine agonistic drugs, which either increase dopamine, prevent its breakdown, or mimic dopamine inside the brain. These medications include Carbidopa-Levodopa, Entacapone, Pramipexole, Ropinerole, Selegiline, and others. However, long-term usage of dopamine drugs for Parkinson's Disease can lead to the formation of dyskinesia and psychosis. Parkinson's Disease patients generally have a wide variety of prognoses, depending on the severity of symptoms, the age of onset, the complications associated with treatments, and the quality of life.

## 3. Gluten as an agent of inflammation

Gluten is a complex protein found in wheat, barley, and rye. It is composed of gliadin and glutenin proteins and is a member of the prolamin superfamily of proteins. Proteins within this superfamily are known for their repetitive glutamine and proline sequences and are thought to make gluten water insoluble [18]. Gluten is also known for its potential to trigger inflammation in individuals with gluten sensitivities or CD. Gluten can activate an immune response in susceptible individuals when consumed, thus leading to the release of inflammatory cytokines [19]. These cytokines include TNF-α, IL-1β, IL-6, and IL-8, which are signaling proteins that promote inflammation [19]. In CD, gluten consumption damages the intestinal lining and increases the gut permeability, thus allowing undigested gluten peptides to enter the bloodstream and heighten the immune activity [20].

This inflammatory response extends beyond the gut. Some studies suggest that gluten can contribute to systemic inflammation and impact various tissues throughout the body, including the CNS. The phenomenon of “molecular mimicry” plays a role here. In this specific scenario, gluten peptides share structural similarities with certain neural proteins such as Neuronal Synapsin 1 [21]. This similarity can lead to immune cross-reactivity, which is when the immune system mistakenly attacks neural tissue by mistaking it for gluten peptides [21]. This process can result in neuroinflammation, which has been linked to various neurological conditions such as Parkinson's Disease [22],[23]. Additionally, the triple-hit hypothesis of alpha-synucleinopathy postulates that nutritional sources of alpha-synuclein or cross-reactive nutritional peptides, such as gluten, might enter the oropharynx, thus leading to some alpha-synuclein entering the brain through the olfactory bulb. Additionally, alpha-synuclein may continue to the stomach and intestines; if improperly digested or modified, it can induce the aggregation of alpha-synuclein protein and lead to gut dysbiosis and the delivery of aggregated protein to the brain via the vagal nerve. Finally, gastrointestinal inflammation may lead to a leaky gut and induce breakdowns in the BBB leading to the three hits, which may result in the formation of alpha-synuclein plaques and Lewy Bodies in Parkinson's Disease [22].

In the literature, gluten has been directly implicated in causing some of the issues related to neuroinflammation and Parkinson's Disease. Several studies have suggested that gluten sensitivity and CD may play a role in the pathophysiology. For instance, a study by Mohan et al. (2020) demonstrated that individuals with CD and gluten sensitivity may have an increased activity of tTg-mediated glutamine deamination, which may lead to alpha-synuclein plaques and neurodegeneration in Parkinson's [24]. Additionally, a study by Chao et al. (2020) found that gluten exposure could exacerbate neuroinflammation, which is a hallmark of PD, by triggering an immune response in the gut [25]. This may extend to the CNS and cause damage to the dopaminergic neurons. Furthermore, genetic studies have shown that certain HLA-DQ2 and HLA-DQ8 alleles, which are associated with gluten sensitivity, are also prevalent in PD patients, thus suggesting a potential genetic link between gluten intolerance and Parkinson's development. Therefore, the connection between gluten sensitivity and neurodegeneration is mediated by neuroinflammation and CNS damage.

Chronic inflammation in the CNS can lead to neuronal damage, disrupt the neurotransmitter balance, and may impair cognitive functions [26]. Furthermore, neuroinflammation due to gluten has been linked to other diseases. One example is the reduction in the observed incidence of schizophrenia in populations who consume little to no grains compared to those consuming grain-rich diets. Patients with schizophrenia have markedly high levels of inflammation, and a gluten-free diet (GFD) has been shown to improve both psychiatric and gastrointestinal symptoms in these patients [27]–[29]. Neurological issues such as cerebellar ataxia, peripheral neuropathy, cognitive impairment, and neuropsychiatric diseases have also been associated with CD and an inflammatory response to gluten, thus suggesting the possibility of gluten-mediated inflammation playing a role in the loss of the BBB integrity leading to other neurological symptoms [29]. Psychiatric diseases such as mood disorders (bipolar disorder, major depressive disorder, and anxiety) are also commonly observed in individuals with CD and Non-Celiac Gluten Sensitivity, with bipolar disorder being 17 times more likely to affect individuals with CD relative to the general population [30],[31]. Therefore, gluten can cause neurological damage through its activation of the immune system and nervous system damage, which can lead to a variety of problems. Understanding gluten's impact on the CNS is essential for better patient outcomes in these neurological diseases. Reducing dietary gluten in sensitive individuals could potentially lessen the inflammatory effects on the CNS and influence neurological health.

## 4. Gluten sensitivity, intolerance, and allergy

Gluten-related disorders (GRDs) are conditions caused by the ingestion of gluten, the structural protein primarily found in wheat, rye, and barley. GRDs encompass a spectrum of adverse reactions and are categorized into three main types: gluten sensitivity, CD, and gluten allergy. Understanding the distinction between these conditions is essential in elucidating their significance and potential implications for Parkinson's Disease [32].

### 4.1. Gluten allergy

A gluten allergy is an adverse immunologic reaction that occurs between minutes and hours following gluten exposure [33]. It is commonly categorized into immunoglobulin E (IgE)-mediated and non-IgE-mediated reactions. In IgE-mediated cases, a gluten exposure triggers type 2 T helper cell activation and IgE production. The reaction can manifest as rapid onset symptoms, affecting the skin (e.g., itching, pruritus, swelling), gastrointestinal (GI) tract (e.g., abdominal pain, nausea, diarrhea), nervous system (e.g., foggy thinking, headache), and respiratory tract (e.g., wheezing, shortness of breath). An example is Baker's asthma, which is a common occupational allergy marked by respiratory complications from inhaling wheat flour. The diagnosis of a gluten allergy typically involves a skin prick or lab tests to identify IgE antibodies in the body [32].

On the other hand, a non-IgE-mediated gluten allergy primarily affects the gut, with some involvement in the skin and lungs. Among the causative conditions are food protein-induced enterocolitis syndrome (FPIES), food-protein enteropathy (FPE), and food protein-induced allergic proctocolitis (FPIAP). The diagnosis is primarily based on a clinical presentation, as no biomarkers exist for these conditions [34]. A gluten allergy may play a role in the development of Parkinson's Disease through the increased inflammatory response and IgE-mediated inflammation of the body. This inflammatory state is thought to lead to the weakening of the BBB and inflammation of the CNS.

### 4.2. Celiac disease

CD is a chronic autoimmune reaction to gluten that gradually develops over weeks to years after gluten exposure. Genetic factors play a crucial role in CD development, particularly human leukocyte antigen (HLA) class II genes (HLA-DQ2, HLA-DQ8). While the presence of these genes is necessary, it is not sufficient alone to estimate the susceptibility. The diagnosis involves specific and sensitive serological tests, such as the anti-tissue transglutaminase (tTG) and IgA anti-endomysial antibody (EMA) tests. Patients with elevated tTG and EMA levels typically exhibit celiac enteropathy and small intestine damage on biopsies. The symptoms of CD widely vary and include both intestinal (e.g., diarrhea, abdominal pain) and extra-intestinal manifestations (e.g., anemia, osteoporosis, neurological symptoms). Importantly, neurological disturbances and disorders, including mood disorders and cognitive dysfunction, have been observed in CD patients, thus suggesting a possible systemic inflammatory link to gluten exposure [33].

### 4.3. Gluten sensitivity

Gluten sensitivity, particularly non-celiac gluten sensitivity (NCGS), is immune-mediated, emerging hours to days after gluten exposure. These conditions are unique in that patients experience adverse reactions to gluten despite the absence of allergic and autoimmune mechanisms. Rather, the innate immune activation is believed to play a role in its pathophysiology. Symptoms of NCGS include abdominal pain, diarrhea, weight loss, bloating, and fatigue. Recent studies have suggested that additional wheat ingredients besides gluten may contribute to NCGS development. The diagnosis of this condition relies on a clinical presentation and the exclusion of gluten allergy and CD [32]. Similarly, a gluten allergy may lead to an increased risk of Parkinson's Disease. While research on the direct link between a gluten allergy and Parkinson's disease is limited, some studies suggest a possible link. For example, a recent case report described the recovery of a 75-year-old PD patient after following a gluten-free diet for three months. This study further highlights the need to test and explore further the prevalence of CD and non-celiac gluten sensitivity in individuals with Parkinson's Disease [35].

## 5. The gut-brain axis

The gut-brain axis is a communication system that allows for crosstalk between the enteric and CNS. The connection between the autonomic nervous system, nerves of the gastrointestinal tract, and other humoral and metabolic networks allows the brain to influence activities which occur in our gut [36]. While it has been widely accepted that our neuro-functionality can influence several different aspects downstream of our body, there is a need to explore how downstream bodily components influence the brain and its physiological function. The health of our gastrointestinal tract can change throughout life. The most pertinent changes drastically occur during the first year of life when the microbial population colonizes and populates its host's gut [37]. Once the solidification of the gut microbiome is established after the first year of life, the health of the gastrointestinal tract can be influenced throughout one's life by several environmental factors such as diet, medication use, aging, as well as psychological factors such as stress [37]. This leads to the importance of inflammatory molecules such as gluten in affected individuals and their ability to cause damage and possibly lead to PD.

The microbial population of the enteric system plays a role in the gut-brain axis and its function. The investigation of several different microbes commonly found in the gastrointestinal tract has found that they play a role in modulating the effects of the immune response. *Bacteroides fragilis*, which is a commensal bacterial found in the gastrointestinal tract and mucosal surfaces of humans, was found in mouse models to modulate the T-cell dependent immune response by producing Polysaccharide A, which is a product which downregulates intestinal inflammation [38]. Another study showed that other microbial genera beyond Bacteroides, such as Odoribacter and Butyricimonas, have a role in immunomodulatory and anti-inflammatory effects by producing short-chain fatty acids that decrease the BBB permeability. The importance of these genera was proven again through this study, as it was found that the Bacteroides, Odoribacter, and Butyricimonas genera were all found in decreased quantities in the gut microbiota of patients with Alzheimer's [39]. This shift in the microbial populations and their connection to inflammation of the BBB may also possibly occur with gluten and Parkinson's Disease. Studies have found that gut inflammation can influence brain function, with early PD symptoms often including gastrointestinal issues such as constipation [40]. These findings underscore the importance of gut health in PD and suggest that focusing on the microbiome could offer possible therapeutic avenues for managing the disease in the acute and chronic phase.

There is no question that the gut-brain axis plays a significant role in how our brain functions. It cannot be denied when looking at the effects of gut microbial dysbiosis on several neurodegenerative disorders such as Alzheimer's Disease and Parkinson's Disease [39]. The trend between the presence of neurodegenerative disorders and increasing gastrointestinal dysfunction shows that there may be an implication of the gut as a more prominent player in the onset of neurological disorders. The Ascending Anatomical Theory asserts that cognitive decline and the neurological pathology develop due to the significant effect the gut has on the brain [41]. Relevant studies show that 80% of patients with PD have accompanying gastrointestinal issues. Additionally, other studies have detected significant populations of patients with both Parkinson's Disease and Inflammatory Bowel Syndrome, which show how prominent the gut's role is in this axis [42]. The interconnectedness of the gastrointestinal tract and the CNS, as demonstrated by the gut-brain axis, shows how closely each of these systems can affect each other and possibly lead to neuroinflammation and damage of the CNS.

## 6. Gluten's effect on the gut microbiota

The intestinal microbiome has become a relevant topic of interest for an individual's health status and as an environmental factor for the development of various immunologic diseases not explained by genetics. The idea of gut microbiota has also begun to be more closely studied in its relationship to Parkinson's Disease. Dietary intake greatly influences the microbiome in both composition and function, which itself regulates homeostasis and metabolism. Firmicutes, Bacteroidetes, and Actinobacteria are the three major phyla that compose the microbiome. Dysbiosis is associated with several chronic gastrointestinal (GI) diseases, such as CD and inflammatory bowel disease [43]. It is simply the decrease of certain species of bacteria and the overgrowth of others. The overgrowth can cause constipation, bloating, and inflammation within the gut, which is thought to carry over to the CNS.

The gluten protein contains high amounts of proline, which leads to incomplete digestion as human GI enzymes do not contain prolyl endopeptidase. As a result, the undigested gluten promotes a pro-inflammatory interaction with GI epithelium and mucosa. Additionally, the high gluten peptides promote proteolytic bacteria growth and the subsequent development of dysbiosis [43]. Dysbiosis is thought to lead to a possible autoimmune activation via post-translational modifications of gluten protein [44].

In CD, gluten conditions the microbiota via an increase in the total number of bacteria, as well as a decrease in the protective (Bifidobacteria, Firmicutes, Lactobacilli, Streptococcaceae) to harmful bacteria (Bacteroides, Prevotellla, E. Coli, Serratia, Klebsiella, Haemophilus, Bacterioidetes, Proteobacteria) ratio [45]. Recent studies have postulated that the inflammation caused by alterations in the gut microbiota can cause neuroinflammation via the microbiota-gut-brain axis [46]. Additionally, recent studies have compared the microbiomes of Parkinson's patients to healthy patients: they found that a reduction in Prevotellaceae and an increase in Bacteroides and lipopolysaccharides from other Gram-negative bacteria were associated with an increased severity of motor symptoms [47]–[49]. Therefore, the changes in the gut microbiome can lead to neuroinflammatory changes via the gut-brain axis and lead to the possible development of Parkinson's Disease in a chronically inflamed state with dysbiosis. Further studies looking at specific bacteria colonies and their prevalence in PD patients may be helpful to elucidate the effect certain bacterial overgrowths can have on PD.

## 7. Blood brain barrier inflammation and gluten

The BBB is an important structural component of the brain that prevents neurotoxic components from infiltrating the CNS. It is composed of choroid plexus epithelial cells, which produce cerebrospinal fluid that flows throughout the brain via ventricles. It strictly regulates what molecules, ions, and chemicals can enter. Thus, it has an important role in maintaining CNS homeostasis as well as protecting the brain from pathogens, injury, disease, or inflammation [50].

Several CNS disorders can have a leaky BBB, thus leading to unwanted infiltrates within the CNS. These diseases include multiple sclerosis (MS), strokes, epilepsy, traumatic brain injuries, Alzheimer's disease, and many others. This occurs due to breakdowns or issues in the BBB's tight junctions, transporters, or leukocyte adhesion molecules (LAM) [50]. The BBB dysfunction which occurs within these disorders, is often secondary to the disease but may lead to an explanation of some of the symptoms presented in each disease. Emerging research indicates that the BBB is also compromised in individuals with Parkinson's Disease. Some studies have found that Parkinson's Disease patients have increased blood-cerebrospinal fluid levels of the albumin protein, which suggests barrier leak [51]. Therefore, the connection between gluten and inflammation and damage to the BBB becomes essential to further understand.

With gluten being an inflammatory molecule that can lead to systemic inflammation, some studies have hypothesized a mechanism in which the BBB becomes leaky to patients due to excessive gluten consumption. This hypothesis has been called the “leaky gut, leaky brain” hypothesis, where inflammation in gluten-sensitive or intolerant populations can lead to the production of inflammatory molecules that not only affect the gastrointestinal tract but also travel to the brain via communication within a system known as the microbiota-gut-brain (MGB) axis. The MGB axis is the term used to define the system that connects the CNS to the enteric nervous system (ENS) via the vagus nerve [52],[53].

**Figure 1.**
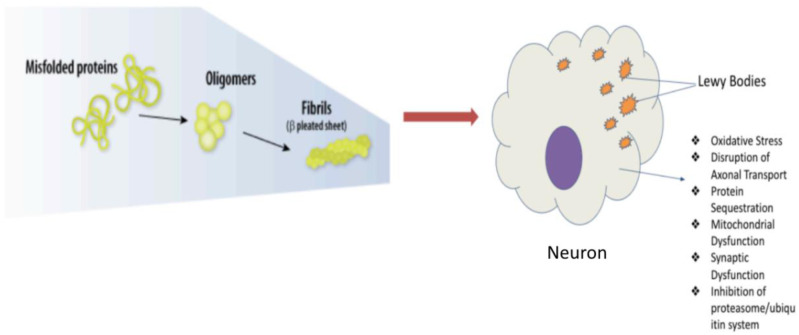
Proposed mechanism of gastrointestinal-derived misfolded proteins inducing apoptosis and metabolic dysfunction in the Substantia Nigra.

As mentioned, findings have suggested that excessive gluten consumption has led to the flattening of the gut villi and poor absorption of nutrients within the body. The mechanism by which this occurs is suggested to result from an increase in cytolytic granules from lymphocytic Fas/FasL ligand interactions [23]. Along with the gut-villi flattening for patients with CD or non-celiac wheat sensitivity, more recent studies have suggested that gluten may play a role in neurodegeneration [54]. Specifically, different studies suggest that the MGB axis plays a role in these deficits. With the inflammation that arises in the gut villi for patients with CD or non-celiac wheat sensitivity patients, the authors hypothesize that the inflammatory molecules may travel systematically within the body and the MGB axis [9]. These inflammatory molecules may induce a breach in the BBB, thus leading to neurological deficits within patients that are infected [9],[54]. This proposed hypothesis model, as referenced in Figure 1, may shed light on how gluten and chronic inflammation may lead to damage of the BBB and the development of neuroinflammation and CNS damage in Parkinson's Disease.

## 8. Conclusions

Parkinson's Disease is a neurodegenerative demyelinating disease that leads to the development of several motor and non-motor symptoms and occurs due to neuronal cell death of the Substantia Nigra. Parkinson's Disease is commonly seen in elderly patients, although it does sometimes show up in younger patients, and this is thought to be due to genetic causes. Additionally, Parkinson's Disease has been linked to several environmental causes and toxin exposures, such as MPTP, trichloroethylene, and heavy metal exposure. It is thought that exposure to these harmful compounds over time leads to the development of Parkinson's Disease. A similar model is thought to occur for gluten and Parkinson's Disease, where long-term exposure and the intake of gluten in patients who are allergic or sensitive can lead to increased inflammation and the production of the disease.

Gluten-related inflammation can lead to possible damage to the brain via the gut-brain axis. Cytokines such as TNF-α, IL-1β, IL-6, and IL-8 may promote inflammation. In CD, gluten consumption damages the intestinal lining and increases gut permeability, thus allowing undigested gluten peptides to enter the bloodstream and heighten the immune activity. This inflammatory response can extend beyond the gut and possibly lead to other diseases such as Parkinson's Disease. Gut microbiota are also an important part of gluten breakdown and inflammation, and may be implicated in the production of increased inflammation. A hypothesis of leaky gut from inflammation of cytokines, microbiota, and gluten inflammation may also lead to further increased inflammation. These factors together may lead to the possible production of Parkinson's Disease presentation.

The relationship between gluten and Parkinson's Disease has gained attention in recent years, as studies suggest that gluten may play a role in the development or exacerbation of neurodegenerative conditions such as Parkinson's. While gluten itself does not directly cause Parkinson's Disease, its potential to trigger autoimmune responses and inflammation in gluten-sensitive or intolerant individuals could lead to the onset or progression of the disease. Therefore, understanding this connection is crucial to increasing research into targeted therapies and preventative measures for those at risk. Further studies should focus on the level of inflammation gluten can cause as well as the areas of the brain and body which are significantly damaged from this inflammation. Furthermore, tests can be performed to elucidate the biomechanisms in the production of alpha-synuclein plaques and their relations to gluten and inflammation.

It is also important to keep in mind that some studies have found no significant association between gluten intake and the risk of Parkinson's Disease. While there is ongoing research exploring the potential link between gluten and neurodegenerative disorders, the evidence remains mixed. Some studies suggest that other factors, such as genetic predisposition, environmental exposures, or other dietary components, might play a more prominent role in the development of Parkinson's Disease. However, the complex nature of Parkinson's Disease and individual variabilities in response to gluten makes it challenging to draw definitive conclusions, and more research is needed to fully understand any potential connections.

This paper explored how gluten can lead to changes in gut microbiota and how these changes can damage the brain via the gut-brain axis. The gut-brain axis is the neural connection between the intestines and the CNS. Excess inflammation can lead to damage to various structures within the brain, including the Substantia Nigra. Further studies should look at the connection between inflammation from various food products and their link to the production of Parkinson's Disease. Additionally, studies looking at the reasons why gluten leads to inflammation and the activation of the immune system within the human body are further warranted.

## Use of AI tools declaration

The authors declare they have not used Artificial Intelligence (AI) tools in the creation of this article.
